# Pleuritic Chest Pain as an Initial Presentation of Pyogenic Spondylitis

**DOI:** 10.7759/cureus.82501

**Published:** 2025-04-18

**Authors:** Keisuke Kuroda, Hirofumi Nakazaki, Yuri Shibata, Hiroki Ikeda, Akira Yamasaki

**Affiliations:** 1 Department of Respiratory Medicine, Tottori Red Cross Hospital, Tottori, JPN; 2 Division of Respiratory Medicine and Rheumatology, Department of Multidisciplinary Internal Medicine, Faculty of Medicine, Tottori University, Yonago, JPN; 3 Division of Orthopedic Surgery, Department of Sensory and Motor Organs, Faculty of Medicine, Tottori University, Yonago, JPN

**Keywords:** mrsa, pleural effusion, pleurisy, pyogenic spondylitis, staphylococcus aureus

## Abstract

Pyogenic spondylitis typically presents with back and neck pain, fever, and fatigue. Sharp chest pain upon deep breathing, commonly associated with pleurisy, is not a typical manifestation of pyogenic spondylitis. We report a case of pleuritic chest pain as the initial presentation of pyogenic spondylitis. A 62-year-old male patient presented with a one-week history of right-sided pleuritic chest pain and fever. Chest computed tomography (CT) revealed a right pleural effusion. The patient was initially treated for bacterial pleurisy without improvement and subsequently developed back pain. Blood cultures yielded methicillin-resistant *Staphylococcus aureus *(*S.* *aureus*​​​​). Additional thoracic spine magnetic resonance imaging (MRI) demonstrated pyogenic spondylitis with abscess formation on the right side of the vertebral body. The final diagnosis was pyogenic spondylitis that had progressed and spread inflammation to the pleura. This case highlights that pyogenic spondylitis can cause secondary pleurisy due to extension of inflammation to adjacent structures, a possibility that should be recognized. When *S. aureus* bacteremia is detected during the course of pleurisy, clinicians should consider secondary pleurisy and perform imaging studies to evaluate for pyogenic spondylitis.

## Introduction

Pyogenic spondylitis is a spinal infection commonly caused by bacteria spreading through the bloodstream. The most common causative organism is *Staphylococcus aureus *(*S. aureus​*​​​​​), which accounts for more than half of the cases. Other pathogens include streptococci, gram-negative bacteria, and *Mycobacterium tuberculosis*, which may also cause vertebral infection [1,2]. Typical symptoms include back or neck pain, along with nonspecific signs such as fever, nausea, or fatigue [3]. The differential diagnosis includes tuberculous spondylitis, metastatic spinal tumors, and noninfectious spinal conditions. Magnetic resonance imaging (MRI) is useful for identifying spinal infection and assessing its extent. Blood cultures are positive in approximately 50% of cases and are helpful for identifying the causative organism. In culture-negative cases, image-guided biopsy is recommended to help distinguish infection from tuberculosis or malignancy [1,4]. Treatment usually requires at least six weeks of antibiotic therapy. Surgical intervention is indicated in cases of neurological compromise, spinal instability, or failure of medical therapy. Despite appropriate management, some patients experience persistent pain or neurological sequelae [5,6].

Pleurisy is inflammation of the pleura, the membrane surrounding the lungs, and typically causes sharp chest pain that worsens with deep breathing. It is usually caused by bacterial or viral infections but can also occur secondary to inflammation extending from nearby organs. Although rare, pyogenic spondylitis can result in secondary pleurisy or empyema when infection spreads beyond the spinal column [7,8]. Such cases are uncommon and may present with pleuritic chest symptoms rather than the typical back pain, leading to diagnostic delays. Herein, we report a case of pyogenic spondylitis presenting with pleuritic chest pain as the initial symptom, highlighting the importance of considering this atypical presentation in the differential diagnosis.

## Case presentation

**Figure 1:**
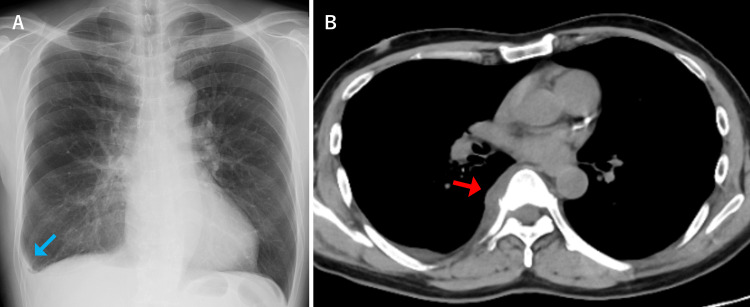
Chest radiography and plain chest CT images on initial examination CT: computed tomography (A) Chest radiography shows blunting of the right costophrenic angle, suggesting a small amount of pleural effusion (blue arrow). (B) Chest CT shows mild soft tissue density on the right side of the T8-9 vertebral bodies (red arrow)

A 62-year-old male patient with a history of atopic dermatitis presented with a one-week history of right-sided pleuritic chest pain and fever. At the time of presentation, his vital signs were as follows: blood pressure of 136/74 mmHg, heart rate of 92 beats per minute, temperature of 37.8°C, and SpO_2_ of 96% on room air. Physical examination revealed no significant findings, and his breath sounds were normal. Laboratory tests showed a white blood cell count of 7,510/μL and a C-reactive protein (CRP) level of 14.09 mg/dL, indicating an elevated inflammatory response. Chest radiography demonstrated blunting of the right costophrenic angle, suggesting a small amount of pleural effusion, but not enough to perform thoracentesis (Figure 1A). Chest computed tomography (CT) showed no abnormalities in the lung fields, only a small right pleural effusion. Additionally, a mild soft tissue density was noted on the right side of the T8-9 vertebral bodies, but its clinical significance was unclear at this point, as the patient had no complaints of back pain (Figure 1B). Although the initial diagnosis was not definitive, bacterial pleurisy was considered the most likely cause based on the clinical presentation. Therefore, treatment with oral amoxicillin-clavulanic acid was initiated to observe the response to antibiotics. However, no improvement was observed, and back pain subsequently developed. Eighteen days after the initial presentation, the patient was admitted for further investigation and treatment. 

**Figure 2:**
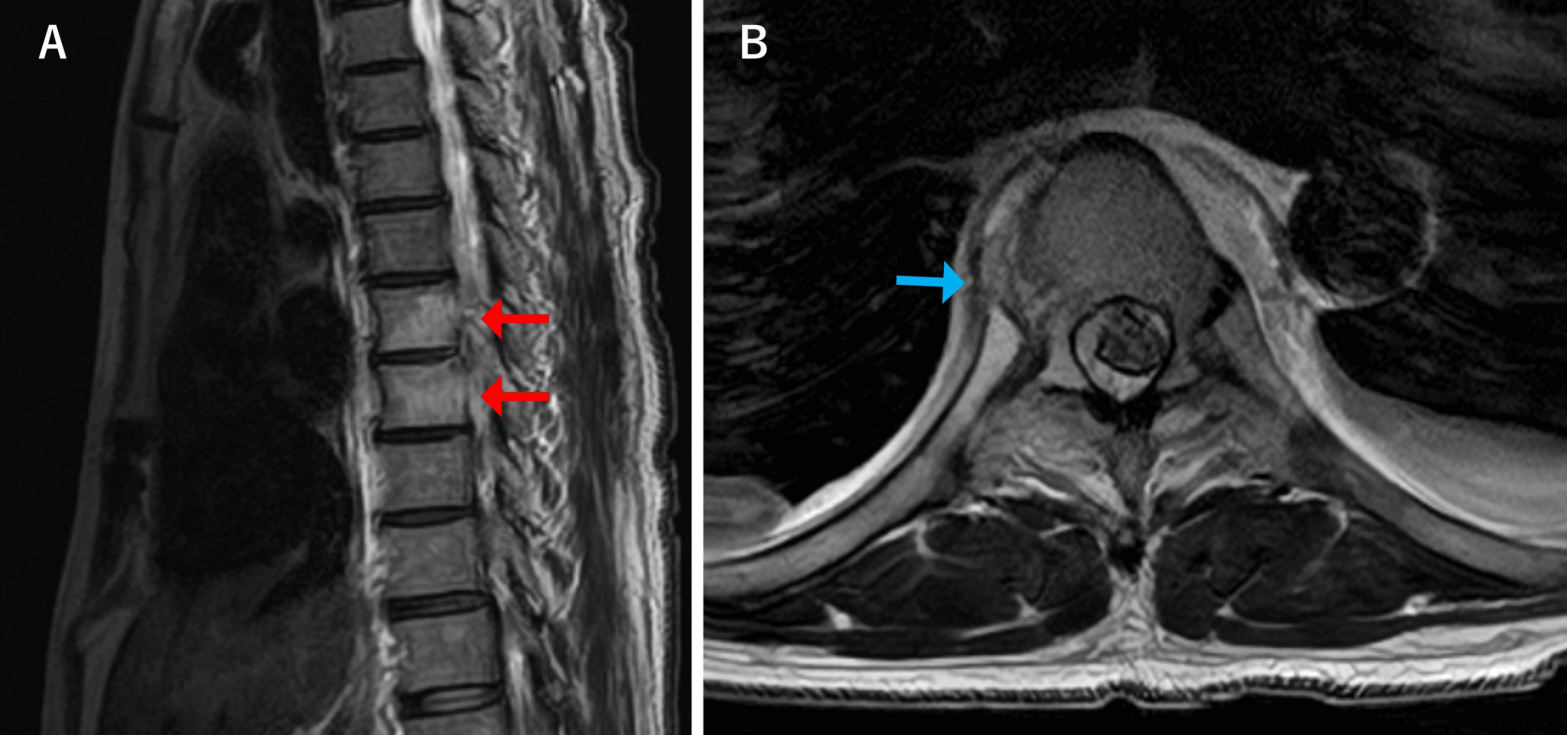
MRI images on the second day after admission MRI: magnetic resonance imaging (A) T2-weighted sagittal image shows a high signal intensity in the bone marrow of the T8-9 vertebral bodies (red arrows). (B) T2-weighted axial image shows a high signal area on the right side of the vertebral body at the T8-9 level (blue arrow)

**Figure 3:**
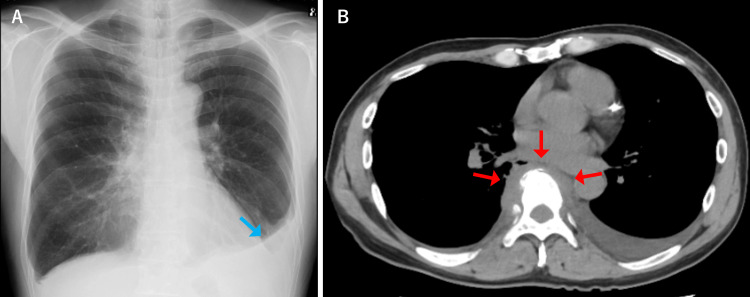
Chest radiography and plain chest CT images on the 22nd day after admission CT: computed tomography (A) Chest radiography shows blunting of the left costophrenic angle, suggesting the appearance of a left pleural effusion (blue arrow). (B) Chest CT shows extension of the soft tissue density around the vertebral bodies to the left side (red arrows)

Upon admission, two sets of blood cultures were obtained, and intravenous sulbactam/ampicillin treatment was started. On the second day of hospitalization, gram-positive cocci suspicious for *S. aureus* were detected in the blood cultures, and the antibiotic was changed to vancomycin (VCM). As the detection of* S. aureus* in blood cultures is uncommon in the course of bacterial pleurisy, and since there was also the appearance of back pain, MRI of the thoracic spine was performed, revealing findings suggestive of pyogenic spondylitis at the T8-9 level (Figure 2A). A T2 high-signal area was also observed on the right side of the vertebral body at the same level, suggesting inflammatory spillover to the paravertebral body (Figure 2B). These findings were consistent with early osteomyelitis, characterized by bone marrow edema and adjacent soft tissue involvement. The gram-positive cocci were confirmed to be methicillin-resistant *S. aureus* (MRSA), and VCM was continued. By hospital day 14, the CRP level had decreased to 2.00 mg/dL. However, on day 21, the patient developed new-onset left-sided pleuritic chest pain, and the CRP level increased to 7.04 mg/dL. A follow-up chest radiography and CT on hospital day 22 showed an extension of the soft tissue density around the vertebral bodies to the left side and the appearance of a left pleural effusion (Figure 3). It was concluded that the pyogenic spondylitis had progressed to a paravertebral abscess, causing the spread of inflammation to the pleura and resulting in pleurisy symptoms. Left thoracentesis was performed, and pleural fluid analysis showed an exudative effusion with no bacterial growth. VCM treatment was continued, but on hospital day 31, the patient developed weakness in his right lower limb, suggesting nerve compression due to an epidural abscess. Neurologic examination revealed grade 3/5 weakness of the right iliopsoas, decreased sensation to touch and pain below the umbilical level, and brisk patellar and Achilles tendon reflexes. On hospital day 32, he was transferred to a nearby orthopedic hospital for surgical management. After transfer, he underwent a T8-9 laminectomy and T6-11 posterior spinal fusion. MRSA was also detected in the paravertebral abscess. Mycobacterial culture and polymerase chain reaction testing of the abscess were both negative. 

## Discussion

Pyogenic spondylitis is often caused by the hematogenous spread of bacteria [1]. It frequently forms abscesses in the epidural space or paravertebral region, which can compress nerve roots [6]. Delayed diagnosis can lead to neurological complications and bone destruction, making early diagnosis and treatment crucial [5]. Typical symptoms include back pain and neck pain, while other symptoms such as fever, nausea, and fatigue may also be present [3]. In patients with elevated inflammatory markers and complaints of back or neck pain, pyogenic spondylitis should be considered in the differential diagnosis [1]. In this case, pleuritic chest pain was the initial symptom, which was atypical and led to a delayed diagnosis.

Chest pain on inhalation generally suggests inflammation of the pleura [9]. Pleurisy is often caused by bacterial or viral infections, while other causes include malignancies and collagen diseases [9]. It can also occur secondary to the spread of inflammation from surrounding organs, such as liver abscesses or pancreatitis [10]. Although rare, pyogenic spondylitis can cause secondary pleurisy due to the extension of inflammation to the surrounding structures [7,8]. In this case, the patient initially presented with right-sided pleuritic chest pain but later developed left-sided chest pain as well. The initial CT findings showed soft tissue density only on the right side of the vertebral bodies, but as the soft tissue density extended to the left side, the patient developed left pleural effusion and left-sided chest pain. This suggests that the pyogenic spondylitis progressed to a paravertebral abscess, causing the spread of inflammation to the adjacent pleura. Spinal MRI is the most recommended imaging test in patients with suspected spondylitis [1]. Spinal MRI was added in this case as well, leading to the diagnosis. In this case, image-guided aspiration biopsy was not performed because blood culture detected *S. aureus*, but aspiration biopsy is recommended if blood culture did not detect the causative organism [1].

Pleural infections often occur secondary to bacterial pneumonia [10]. The most common causative organisms include *Streptococcus pneumoniae*, *Haemophilus influenzae*, *Moraxella catarrhalis*, and anaerobic bacteria [10,11]. *S. aureus* is also an important causative organism, particularly in nosocomial infections. The blood culture positivity rate in respiratory infections is low [12], and when *S. aureus* is detected in blood cultures, it is generally necessary to investigate the presence of intravascular catheters or other medical devices and the presence of skin and soft tissue infections [13]. Moreover, *S. aureus* bacteremia often causes metastatic foci, and the presence of infectious endocarditis, spondylitis, or abscess formation should be evaluated [13]. This patient did not have any medical devices or skin and soft tissue infections but had a habit of scratching the skin due to atopic dermatitis. It has been reported that patients with atopic dermatitis are more susceptible to *S. aureus* skin colonization and bloodstream infections due to impaired skin barrier function [14]. It is presumed that the patient developed a bloodstream infection through the skin as the portal of entry and subsequently developed pyogenic spondylitis via hematogenous spread. It is a basic principle that obtaining cultures before initiating antimicrobial therapy is extremely important for determining the treatment strategy in infectious diseases. If *S. aureus* is detected in blood cultures during the course of pleurisy, the presence of pyogenic spondylitis should be considered.

## Conclusions

We reported a case of pyogenic spondylitis presenting with pleuritic chest pain as the initial symptom. Pyogenic spondylitis can cause secondary pleurisy due to the spread of inflammation to the surrounding structures, and this possibility should be recognized. When *S. aureus* bacteremia is detected during the course of pleurisy, secondary pleurisy should be considered, and imaging studies should be added with pyogenic spondylitis in mind.
